# With a grain of salt? Supervisor credibility and other factors influencing trainee decisions to seek in-consultation assistance: a focus group study of Australian general practice trainees

**DOI:** 10.1186/s12875-020-1084-7

**Published:** 2020-02-07

**Authors:** Nancy Sturman, Christine Jorm, Malcolm Parker

**Affiliations:** 1 Primary Care Clinical Unit, Faculty of Medicine, The University of Queensland, Brisbane, Queensland Australia; 2 New South Wales Regional Health Partners, Newcastle, Australia; 3 Faculty of Medicine, The University of Queensland, Brisbane, Australia

**Keywords:** General practice training, Clinical oversight, Help-seeking, Expertise

## Abstract

**Background:**

‘Ad hoc’ help-seeking by trainees from their supervisors during trainee consultations is important for patient safety, and trainee professional development. We explored trainee objectives and activities in seeking supervisor assistance, and trainee perceptions of the outcomes of this help-seeking (including the utility of supervisor responses).

**Methods:**

Focus groups with Australian general practice trainees were undertaken. All data was audio-recorded and transcribed, coded using in-vivo and descriptive codes, and analysed by the constant comparison of provisional interpretations and themes with the data. Findings are reported under the over-arching categories of help-seeking objectives, activities and outcomes.

**Results:**

Early in their general practice placements trainees needed information about practice facilities, and the “*complex maze*” of local patient resources and referral preferences: some clinical presentations were also unfamiliar, and many trainees were unaccustomed to making patient management decisions. Subsequent help-seeking was often characterised informally as “*having a chat*” or “*getting a second opinion*” so as not to “*miss anything*” when trainees were “*not 100% sure*”. Trainees emphasised the importance of being (and demonstrating that they were) clinically safe. Workflow constraints, and supervisory and doctor-patient relationships, had a powerful influence on trainee help-seeking activities. An etiquette for providing help in front of patients was described. Trainees assessed the credibility of supervisors based on their approach to risk and their clinical expertise in the relevant area. Several trainees reported reservations about their supervisor’s advice on occasions.

**Conclusion:**

A trainee’s subsequent help-seeking is strongly influenced by how their supervisor responds when their help is sought. Trainees prefer to seek help from credible supervisors who respond promptly and maintain trainee ‘face’ in front of patients. Trainees learn through help-seeking to make their own clinical decisions but may remain uncertain about professional and societal expectations, and curious about how other general practitioners practise. Trainees value opportunities throughout their training to observe expert general practice.

## Background

Following the completion of hospital-based training requirements (a minimum of two years), Australian general practice trainees work for three 6-month terms (Terms 1,2 and 3) across two accredited training practices, and complete a final 6 month advanced training term (Term 4) in either a general practice or hospital post. Trainees are able to practise without direct supervision of every clinical encounter from their first general practice term [1–3], although the supervisor is expected be onsite and available during office hours for most of the time in Term 1 [3]. Trainees are encouraged to seek help from their general practitioner supervisors before, during and/or after consultations if they are not certain how to manage patients independently [4, 5], and ‘ad hoc’ help-seeking during consultations in particular is believed to contribute to ensuring patient safety and trainee learning and development [5–7]. In-consultation help-seeking by general practitioner trainees is a complex social interaction embedded in the pace and pressure of general practice work. Despite its importance, little is known about the general practitioner trainee perspective on help-seeking decisions, activities and outcomes.

Various conceptual approaches have been used to frame medical trainee help-seeking, including the following theories: expected utility [8]; self-regulation [4]; organisational safety [9, 10]; legitimate participation [11]; and workplace affordances [12].

A subjective expected utility approach to help-seeking in workplaces assumes that help-seekers weigh up the likely costs and benefits in deciding whether and how to seek help [8]. Perceived costs include self-presentation and ego costs [13]. Previous research on help-seeking in hospital clinical training highlights the importance of managing the impressions of peers and supervisors when seeking assistance, and the potential costs of acknowledging uncertainty, disrupting team workflow and/or relinquishing autonomy [14, 15]. An additional cost in general practice help-seeking may be a reduction in patient impressions of trainee competence [7]. There has been less work on the benefits of help-seeking, including the credibility and value of supervisor assistance [16, 17]. There are several reasons to be concerned that contemporary general practice trainees may have more reservations about the credibility and expertise of their supervisors than trainees working in a hospital environment. Trainees may be influenced by the relatively low regard for general practitioner expertise still found in some hospital cultures [18], and supervisors may actually downplay their expertise in their scripts for providing in-consultation assistance to trainees, as they try to promote trainee autonomy and avoid undermining patient impressions of trainee competence [19]. Trainees have limited opportunities to observe the work of their general practitioner supervisors as this is largely invisible to trainees behind the closed doors of supervisor consulting rooms. Trainees may therefore be ambivalent about the value of their supervisor’s greater clinical experience, and the status of local practice norms, particularly if these appear to conflict with widely available, apparently authoritative, guidelines [19, 20].

Self-regulation approaches to help-seeking in clinical training emphasise meta-cognitive processes and constructs, including self-monitoring and self-entrustment. Self-monitoring allows medical practitioners to ‘slow down’ during clinical decision-making in order to engage active attention and avoid errors [20–22], including the premature resolution of clinical uncertainty [23]. Levels of self-entrustment and self-confidence determine whether a general practice trainee decides to complete their consultation independently or seek support from a supervisor [4]. Irrespective of the particular constructs involved, the ability to maintain a “modicum of chronic unease” [23, 24], tolerate uncertainty until a safe resolution is achieved, and respond attentively and flexibly, is an important theme in both the clinical decision-making [21–23] and organisational safety literature [10, 23]. Seeking help is usually viewed from these approaches as being an appropriate response to low levels of confidence and/or persistent uncertainty.

The community of practice approach emphasises the sociocultural context of workplace training, and foregrounds increasingly legitimate participation by the trainee in the work of the community, and tensions between the reproduction and the transformation of practice [11] as the new displaces the old. Trainee help-seeking may be viewed from this perspective as functioning to secure trainee legitimacy, gain access to the affordances of the workplace [12] and negotiate professional identities and tensions.

In this study we explored the general practice trainee perspective on seeking in-consultation help. The aim of this study was to explore trainee objectives and activities when seeking help from supervisors during patient consultations, and their perceptions of the outcomes of help-seeking, including the utility of supervisor responses.

## Methods

The study used focus group discussions to collect data from general practice trainees. Focus groups were chosen to reduce the impact of any social distance between the facilitator and participants on the discussions, and gain more insight into trainee group identity, talk and dynamics [24, 25]. We acknowledge that participants do not have direct insight into their own cognitive processes, and that they fashion their accounts for a particular audience, in view of their self-presentation and other agendas [25, 26]. However we take a constructivist realist position [26, 27] that there are links between these accounts, and internal, external and social worlds which are useful to explore and understand.

Focus groups were held immediately prior to, or following, trainee education release days, at premises rented by the training organisation. Information about the study was provided by medical educators at previous education sessions, who distributed written participant information (including the study focus on trainee help-seeking) to trainees expressing interest in participation. All trainees who contacted the focus group facilitator (the corresponding author) by email to confirm interest took part in a focus group. Sampling was by convenience and no record of non-consenting trainees was kept. Several participants may have recognised the facilitator from her previous role as coordinator of undergraduate medical programme general practice placements, but none had a current relationship. Focus group participants were known to each other, as they attended the same small groups for their trainee education release days. No-one other than the participants and facilitator was present. See Table 1 for the Focus Group Guide, which was not pilot tested.

**Table 1:** GP trainee Focus Group Discussion Guide

1. Issues of confidentiality and anonymity. Reinforce written participant information, emphasising that no participant would be identifiable in any dissemination or publication of the study by the investigators. Establish ground rules for participants. Advise participants to draw the group’s attention to any information that they do not wish to be repeated outside the group by other participants in any further discussions. Confirm consent to audio-recording.	
2. Prompts for facilitator What experiences have you had of asking for help from your GP supervisors? (prompt for both clinical and professional practice contexts) How do you decide whether to ask for help from your GP supervisors? Is it difficult to decide whether to ask for help from your GP supervisors? When in particular? How do you ask for help? How do your supervisors prefer you to ask for help? How do you think your supervisors feel about you asking for help? How do you think patients feel about you asking for help? How do you think practice staff feel about you asking for help? Have you had any particularly good experiences of asking for help from GP supervisors? Have you had any particularly bad experiences of asking for help from GP supervisors? (If time permits) What alternative sources of help do you have, and when would you use these?	

The group discussions were experienced by the facilitator as engaged, generous and authentic. All focus group discussion was audio-recorded and transcribed by a professional service, identifying participants by the characteristics of their voice. Handwritten jottings were made concurrently by the facilitator noting comments which warranted further clarification or expansion, or were particularly striking. Transcripts were not returned to participants. Analysis proceeded after all transcripts were read in full by all the authors. Initial coding was performed by the corresponding author, using in vivo and descriptive coding [27, 28], assisted by Atlas.ti software with data management, coding, and queries. Analysis proceeded using a constant comparative approach, to ensure grounding of analysis in the data as initial codes were refined and clustered into higher level codes and developing concepts. An inductive and interpretive approach was used to identify major and minor themes [28, 29]. The authors met twice to discuss their analysis and interpretations of the data and reached a consensus on the overarching themes over multiple re-draftings of this paper.

The corresponding author is a practising general practitioner undertaking a PhD in medical education, CJ is an academic with relevant expertise in patient safety and MP is an academic with expertise in medical ethics, law and professional practice.

## Results

Five focus group discussions (mean duration 43 min, range 35–57) were held in November 2017 with a total of 16 trainees (median group size 3, range 2–5) in Brisbane, Australia. See Table 2 for participant demographic information and Table 3 for focus group membership information.

**Table 2:** Focus group participant demographic information

All participants	
Age	mean 32.7, range 26–47 years
Gender	male 8, female 7
Country of medical qualification	Australia 15, UK 1
Number of years working as a medical practitioner	mean 5.1, range 3–20 years
General practice training term	Term 1 1
	Term 2 10
	Term 3 3
	Term 4 1
Rural or regional general practice experience	Yes 6, No 10
Full-time general practice	Yes (≥8 sessions per week) 9
	No (< 8 sessions per week) 7

**Table 3:** Focus group information: gender, age, training term, years of medical experience^a^

	Focus group 1 (*N* = 3; M1,F2)	Focus group 2 (*N* = 2; M1,F1)	Focus group 3 (*N* = 5; M2,F3)	Focus group 4 (*N* = 4; M2,F1,NR1)	Focus group 5 (*N* = 2; M1,F1)
Participant 1	35,T4,5	31,T2,7	33,T2,3	48,T2,3	28,T1, 3
Participant 2	26,T3,3	36,T2,6	30,T2,4	31,T2,3	47,T2, 20
Participant 3	F,30,T3,5		32,T2,3	27,T2,4	
Participant 4			28,T2,4	NR,T3,4	
Participant 5			29,T2,4		

^a^Gender (M/F), Age (years), GP training term (T1-T4), Medical experience (years worked as doctor)

Not reported (NR)

Findings are discussed below under the three primary aspects of help-seeking which the study aimed to explore: trainee objectives; trainee activities; and trainee perceptions of the outcomes of seeking help. See Table 4 for themes and sub-themes, and Table 5 for illustrative quotations. Trainees’ own words are used at times (indicated by italics between quotation marks) to retain the participant voice in the paper [29, 30] and to draw attention to trainee choice of language in constructing their accounts.

**Table 4:** Trainee help-seeking: themes and sub-themes

Trainee objectives	Trainee activities	Outcomes of trainee help-seeking
1. Managing workflow 1a. The transition 1b. Keeping to time	1. Deferring assistance	1. Scaffolding trainee skills
2. Patient safety	2. Constructing help 2a. Selecting help provider 2b. Presenting the case	2. Managing uncertainty
3. Managing relationships 3a. Relationships with patients 3b. Relationships with supervisors	3. Managing supervisor responses	3. Feedback on help-seeking
4. Professional development

**Table 5:** Trainee help-seeking: Illustrative quotations

**Trainee objectives**	
1a. Managing workflow: Managing the transition to general practice	
P1: Getting to know the ropes of the referral processes and pathology and the computer system as well. . . P2: And just that whole world of community-based services was a bit overwhelming at first.. . where can I get almost like the best deal for patients. . . A lot of it is by word of mouth so talking to the supervisors and seeing what other patients have used. FG2	
1b. Managing workflow: Keeping to time	
P2: But, yeah, sometimes (waiting for the supervisor) can blow my consult out completely. P1: Yeah. And then you’ve got issues around the patient waiting. . . P2: In an ideal world if my supervisor was sitting doing nothing, then I would always get them in to look at the patient, yeah. FG4	
2. Patient safety	
P2: And you want to make sure that you are safe. And that you have shown that you have been safe by discussing with your supervisor about something that you – you are unsure about. Q: Showing to the patient or to the supervisor or? P2: I think to yourself. To the medical legal systems, to the patient and to your supervisor. What everyone wants to know is that you are safe in your practice as a registrar and that, if you are unsure, then you will talk to someone. FG3	
3a. Managing relationships with patients	
P2: I’ll say, ‘I don’t know, exactly, what’s going on here, but I think we could do this and I can talk to somebody more experienced- - - I’ll get you back and we’ll talk about it more’. P1: Yeah, I found a similar thing. Just being able to just say straight up to patients ‘I’m not sure what’s happening, I’m not entirely sure what’s going on’. A lot of the time they actually seem oddly reassured by that [laughs]. .. and they seem to think, ‘oh, I’m getting a second consult’, um, you know, ‘I’m getting a lot of attention’, that’s why I find patients generally like it. FG5	
3b. Managing relationships with supervisors	
My situation’s very different this year and I don’t call for help much at all, and there’s lots of reasons for that. One of them is that I don’t have much of a relationship at all with my supervisor this year. .. they’re not the kind of person that really has much interest in teaching. FG1P2	
4. Professional development	
You kind of are wondering, “What are all these other GPs doing in their room?” You know what I mean? Is what I am doing the standard? And you can look up guidelines and stuff. But so much of what we do – there’s a lot of grey. And so getting a sense of what is a – you know what is – what are my colleagues doing? What is the expectation – both from a legal perspective but also just from a practical, what is the right thing to do, perspective. Um, and I think that is what we need to get a good sense of, in these two years that we do have supervisors available FG2P3	
**Trainee activities**	
1. Deferring assistance	
P2: At first, I would want help here and now all the time. .. so that was (Term 1), I’m now coming to the end of (Term 2). .. I’ll go, ‘Okay. Well, I’m not exactly sure what’s going on but I know it’s not urgent, I’ve ruled out everything serious’. So, I’m going to go away, do a bit of a reading or I’m going to take this case at lunch time and discuss it. So that pattern’s changed. I mean I still ring them here and now, can you come and look at this, but not all the time. I’m confident. And again, I think it’s a confidence thing to say, well, I know this can definitely wait. P1: Yeah, I find a very similar thing FG3	
2a. Constructing help: Selecting a help-provider	
You ask the person who you really trust who’s just going to give you a quick opinion and who’s always bloody right! FG5P4.	
2b. Constructing help: Presenting the case	
It depends on, on the person I am speaking to um, as to what their style is, how they like the registrar interaction to go and you get a feel for that as you work in the practice for longer. Um, but yeah usually everyone appreciates you being direct upfront about what you need. And then showing that you have given some thought to it beforehand P1FG2	
3. Managing supervisor's response	
I think it’s probably the GPs that have been GPs for many, many years, with patients that expect certain things, it’s hard for them to perhaps start to change their practice in a way that’s more in line with antibiotics stewardship. .. the necessity for antibiotics sometimes, you’ll take that really with a grain of salt, and see whether you’re reasonable in not prescribing and having a good return plan FG3P2	
**Outcomes of traineehelp-seeking**	
1. Scaffolding trainee clinical skills	
P3: Yes, you’ve asked for their opinion but they’re just fleshing it out of you. . . P1: And she’s, like, ‘And what else could it be?’.. . And then you, kind of, have had that chance to synthesise your thoughts and in – with basically, like, having a person in your brain going ‘yeah, yeah, you’re doing good, you’re doing good, you’re doing good, yeah, you got it’ FG4	
2. Managing uncertainty	
The thing that one of my supervisors said to me one day when I was asking her about someone. .. she was, like, ‘Well, is she going to die today?’ like, quite blunt and I was, like, ‘No, actually’.. . I could say to this woman, ‘Let’s give this a try and I’ll see you in a week to two weeks and we’ll follow it up’. .. coming to grips with that change in general practice which is that it is a lower acuity, stuff that you do over a longer period of time FG5P1	
3. Feedback on trainee help-seeking	
So I called up my boss and I said like, ‘Would you mind having a feel of her tummy and seeing what you think?’. .. (The supervisor) is like, ‘Well what – what do you think should happen?’ I’m like, ‘I think she should go to hospital.’ He said, ‘Well, that is what should happen I guess.’ In a way kind of saying, “Well, do I really need to see her?”. .. Like I realise that I should be trusting my instincts but in these two years I am here to learn and I am here to get a second opinion about things. .. from a community setting, I think it’s useful. And there was maybe just a little bit of pushback there about something that they felt was an obvious answer FG2P2	

### Trainee objectives

#### 1a. Managing workflow: *Managing the transition to general practice*

Participants consistently reported having sought help frequently during their initial transition from hospital practice into “*the wild safari*” of general practice. Trainees described themselves as “*coming across as really needy*”, “*having no idea*” and being “*overwhelm (ed)”* (see Table 5), indicating their discomfort and uncertainty at this time. Initially trainees needed assistance to obtain information about practice software and other facilities, and the “*complex maze*” of local patient resources and referral preferences. Supervisors, practice staff and patients themselves all contributed to building up this trainee “*database*”. For many trainees, responsibility for patient management decisions, the general practice setting, and some clinical presentations were unfamiliar. Trainees also reported that it was *“comforting”* to have confirmation of their plans in situations which they were encountering for the first time, such as calling an ambulance to transfer a patient to hospital.

#### 1b. Managing workflow: *Keeping to time*

It was common for trainees to contrast this initial stage with being able to considerably reduce their in-consultation help-seeking by the second month of the first term. Trainees still sought in-consultation assistance when they were “*stuck*” in a consultation, but it was clear that time constraints had a major impact on help-seeking decisions, because trainees needed to keep up with patient appointment schedules. Trainees described deferring discussion about patients when either they or their supervisors were busy. When they did seek in-consultation help, they avoided asking supervisors who might unduly delay the trainees’ consultations, and sometimes sought brief advice over the phone (unless sighting the patient was necessary to establish a diagnosis) when they would have preferred a longer face-to-face interaction with the supervisor in the room with the patient, in an “*ideal world”* (see Table 5). Waits of up to 20 min were reported when supervisors completed their own consultation before providing help. Several stories were told of the trainee moving forward with patient management while waiting for the supervisor, risking the embarrassment of having to “*wind back*” plans and “*backpedal*” if the supervisor subsequently changed these.

#### 2. Patient safety

Trainees commonly spoke about their help-seeking after the initial transition as “*getting a second opinion*”, “*double checking*” when they were “*not 100% sure*”, and “*making sure*” that they were “*not missing anything*”. They emphasised the importance of being (and demonstrating that they had been) safe, and frequently talked in terms of being able to “*sleep at night*” only if they were confident that their management was safe.

The clinical contexts of trainee accounts of help-seeking often highlighted concerns about patient safety, including several scenarios in which decisions had to be made about whether to admit a patient to hospital, or rely on patients and their families to monitor for any clinical deterioration. These clinical contexts included febrile children, adults with chest pain and patients with suicidal ideation. Several trainees emphasised that patient safety was always their highest priority (see Table 5).

#### 3a. Managing relationships with patients

Other scenarios for help-seeking which were each mentioned by several trainees included patient requests for drugs of dependence or another clinical opinion, and regular patients of another general practitioner in the practice with ongoing problems. In these scenarios trainee decisions may be particularly subject to criticism by patients and/or colleagues, and help-seeking appeared to be a pre-emptive move to ensure trainee psychological safety.

Many trainees portrayed themselves as having become comfortable managing patient relationships while seeking help. Several reported that although they had been anxious about patient impressions of their competence earlier in their training when admitting gaps in their knowledge, they no longer had these concerns when framing help-seeking as getting a second opinion (see Table 5).

However a few *t*rainees reported losing ‘face’ in front of patients due to supervisor breaches of the etiquette of help provision, a key aspect of which is not undermining patient impressions of the trainee’s expertise. One participant described being “*burned*’ by an abrupt, “*old school”* supervisor who gave simple, direct advice without engaging in any “*chit-chat*” with the patient or exploring trainee concerns. Another participant reported that his ego was “*shredded*” whenever his supervisor provided help, due to what the trainee perceived to be an excessive “*show*” of supervisor expertise to the patient which belittled the trainee’s own competence.

#### 3b. Managing relationships with supervisors

Trainees typically used informal and non-hierarchical language to describe their help-seeking interactions, (“j*ust having a chat”,* and even *“helping each other work out a plan”)*, and their requests of supervisors (*“having a quick look”* at the patient, and *“having a feel of her tummy”,* in contrast to formal medical language (“examining the patient” and “palpating the abdomen”). Their use of this almost off-hand language may have been intended to convey impressions of trainee confidence and status, and to re-frame their help-seeking from a need for assistance from a superior to a casual collegial interaction. Several trainees admitted, however, to being anxious that too much help-seeking would unduly burden supervisors or give the impression that they “*knew nothing*”. Discussions about getting “*back-up*” from “*the boss*”, and comments that supervisors had a role in assessing, and often employing, trainees, also suggested that trainees remained aware of a power differential in the supervisor-trainee relationship.

Trainees described seeking help more frequently when they had a close relationship with their supervisor (see Table 5), irrespective of their stage of training. Trainees in one group reported that other trainees in less accommodating practices than their own avoided asking questions and “*cut corners*” to manage workflow pressures. Another trainee commented that he would have liked his supervisor to “*regularly check in*. *.*. *to kind of say ‘Is this working for you?’*” rather than “*assuming everything is all right*“. These comments suggested that trainees with weaker trainee-supervisor relationships were uncomfortable seeking assistance or initiating these conversations. Several trainees were also reluctant to “*burde*n” other general practitioners in their training practices who were not supervisors, with one trainee explaining that they would be helping “*out of the kindness of their heart*”.

#### 4. Professional development

Trainees talked about being “*here to learn*” and their wish to “*soak in”* as much knowledge as they could, while they were trainees, particularly from supervisors who they perceived to be particularly clinically astute. Several trainees had intentionally selected their training practices because of the good reputation of these supervisors among hospital clinicians, and others also valued opportunities to observe expert clinical practice:

*You know, so he could, you know, look at his patient and sum the whole patient up and work that out, just with that clinical experience. You know, that was right and that was a really useful experience, to actually see that interaction* FG4P2

Trainees described wanting to take opportunities during training to find out more about other doctors’ approaches, what they were “*doing in their rooms*” and how their own practice measured up. These discussions conveyed a sense of trainee isolation and vulnerability, their awareness that their own practice might be exposed to criticism, and a somewhat precarious sense of the “*right thing to do*” (see Table 5). This contrasted with the more confident, casual approach to seeking help which was portrayed elsewhere in the discussions, and positioned the trainees as having legitimate claims on supervisors for assistance.

### Help-seeking activities

#### 1. Deferring in-consultation assistance

Help-seeking typically involved an initial attempt to solve the problem independently, for example by reviewing patient records for previous management strategies or searching online. A more accessible source of advice than the supervisor, such as a practice nurse, was sometimes contacted. If these steps were unsuccessful, a decision was then made about whether help-seeking could be postponed to a more convenient time, in order to reduce the disruption to their own and their supervisors’ consultations and enable more relaxed discussions. Trainees reported becoming confident deferring assistance by using temporising strategies and scheduling patient reviews or contacting outside patient consultations if management changes were suggested at the deferred discussions (see Table 5).

#### 2a. Constructing help: Securing a help-provider

Trainees preferred to seek help from trusted supervisors who were readily accessible (see Table 5). The trainee preference for not interrupting supervisors sometimes led them to wait in corridors and outside consulting or treatment room doors to catch a supervisor in between consultations. Trainees described this activity as “*hovering*” and “*loiterin*g”, using irony to draw attention to the awkwardness and inefficiency involved. If trainees elected to interrupt a supervisor, they typically phoned the supervisor from their consulting rooms.

Several trainees reported that they preferred to distribute their help-seeking to reduce the load on any one general practitioner, and several described “*cherry-picking*” help providers based on perceived areas of provider expertise and their approach to managing risk, where more than one supervisor was available.

#### 2b. Constructing help: Presenting the case

Trainees commonly presented their request for assistance over the phone within the patient’s hearing. However they also reported reasons for preferring to seek help outside the patient’s hearing, including: to avoid information in the case presentation upsetting, worrying or offending the patient; to conceal the extent of the trainee’s uncertainty from the patient; and to avoid the patient overhearing supervisor advice which the trainee might choose not to follow. One participant described “*jumping up*” to leave the consulting room as she noticed the supervisor approaching, in order to speak to the supervisor before he came in.

Trainees reported that the activity of presenting to their supervisor often clarified their thinking, and even just stepping away from the patient allowed trainees to collect their thoughts:

S*ometimes if my head is bursting I will go out, on the pretence of trying to find my supervisor* [laughs]. .. *get my head together* FG4P2

Trainees described supervisors wanting concise and “*direct upfront*” problem presentations (see Table 5) which included a management plan if possible. Most trainees appeared to use medical terminology for these case presentations, although one participant reported using lay terms when the patient was present.

#### 3. Managing supervisor responses

Trainees reported avoiding further in-consultation assistance from supervisors who had breached etiquette or provided poor advice. Several trainees reported advice to prescribe antibiotics which conflicted with their understandings of antibiotic stewardship (although one trainee reported accepting advice to prescribe antibiotics for a patient he had intended to manage conservatively “*because it wasn’t such a bad argument actually”*). Several other trainees reported being surprised by supervisor advice to manage patients at home, instead of admitting them to hospital. A few trainees told cautionary tales of accepting this advice despite misgivings, culminating in the trainee “*chasing up”* patients after hours with abnormal investigation results requiring urgent admission (described by one trainee as “*a big fiasco*”). However other trainees reported witnessing good outcomes from following advice to manage patients at home, and that this had changed their future practice. Several participants referred to the diversity in general practitioner approaches to managing risk and referrals. Supervisors who appeared too ready to refer patients were discussed as well as others who appeared too reluctant:

*My GP*. *.*. *it’s ideology, or whatever it is, sort of has this view of sending patients to hospital is a failure, when he – he thinks he can easily be able to solve everything* FG1P1

A number of stories were also told of unsatisfying advice which seemed to address only one aspect of a more complex problem. Contexts included restricting the prescription of opiate analgesia to an opiate dependent patient, and referring a patient for assessment of their cognitive competence in a situation which appeared to involve the wider issue of financial abuse by an elderly patient’s relative.

Several trainees reported always following their supervisor’s advice, although several others reported having disregarded this on occasions, sometimes after seeking another opinion. One trainee reported that “*what this taught me is that it’s ok to do it differently. That there isn’t always one right answer”.* Disregarding advice was also justified in terms of the trainee having “*to sleep at night*”, and the uneven nature of supervisor expertise, so that some advice should be taken “*with a grain of salt*” (see Table 5). Most trainees who reported having disregarded their supervisor’s advice did appear to find this situation awkward and several reported concealing this from their supervisor.

### Outcomes of trainee help-seeking

#### 1. Scaffolding trainee clinical skills

Although there was a consensus that there was rarely time to teach during in-consultation help-seeking interactions, several trainees reported picking up “*learning points”. T*rainees appeared to appreciate supervisors who talked through their thinking for the trainee’s benefit, and supervisors who used probing questions to scaffold their clinical problem-solving (see Table 5). A “*good teacher*” could provide help efficiently:

*He’s in and out in three minutes but it’s quality, not wanting the whole consult re-done but giving the impression of being unhurried and thoughtful, and he interacts with the patient and reveals his thinking.* FG4P4

#### 2. Managing uncertainty

Many trainees reported coming to accept clinical uncertainty, as something that they had to “*learn to live with*” as general practitioners. Several trainees reported being reassured and impressed when they witnessed their supervisor admitting and managing his or her own uncertainty in front of their patient. They also commented appreciatively on senior general practitioner colleagues who discussed their own cases, with “*no-one’s thinking they know it all”*. Participants reported becoming “*comfortable with uncertainty”,* provided that they knew “*the process*” or “*the steps*” for managing consultations, and “*readjusting (their) perspective*” to solving problems in general practice over a number of consultations (see Table 5). However, several trainees also mentioned seeking help from general practitioners or senior trainees who were “*always bloody right*” or “*very, ridiculously smart*”, suggesting an ongoing belief that it might often be possible to know the ‘answers’.

#### 3. Feedback on trainee help-seeking

Trainees did not report any explicit discouragement from their supervisors to seek help, except for a single mention of audible supervisor sighs on the phone (which seemed to surprise the other trainees).

Some ambivalence was expressed in a number of discussions about supervisors asking trainees to propose a plan and *“trust your judgement*”. On the one hand, trainees were sometimes dissatisfied with “*just hav(ing) to call”* clinical decisions themselves, in several cases framing this response as “*pushback*” (see Table 5). On the other hand, trainees also reported that the encouragement to formulate their own management plans had built up their confidence and that it was appropriate for the trainee to make the management decisions:

*At the end of the day we are their treating practitioner and we still have our supervisor for back up if we need it, but we have been managing this patient*. *.*. *seeing them, like every week or so, we’ve had that rapport, we know more of the history, and the short … sentence, we provide the supervisor with is not necessarily all the stuff that we’ve gained from the patients ... So, I think it’s more that they want us to be confident with our capabilities … rather than ‘push back’.* FG3P1

## Discussion

This study reports on the objectives, activities and outcomes of in-consultation help-seeking from the Australian general practice trainee perspective, building on previous work in Dutch general practice [4]. Previous Australian studies have focused on the clinical context [30, 31] or supervisor style [31, 32] of the in-consultation supervisory encounter, rather than trainee decisions and activities in seeking and securing assistance. Our findings highlight the impact of the psychosocial context of these decisions, including workflow constraints and supervisory and doctor-patient relationships, in addition to the presence of clinical uncertainty. Interestingly, a recent study on general practitioners consulting medical peers for advice highlighted similar factors influencing these decisions [32, 33].

Other new findings include trainee perceptions of, and responses to, the outcomes of help-seeking. Overall trainees portrayed themselves as willing and able help-seekers, who rapidly developed effective strategies to secure any assistance needed to manage patients safely, while minimising disruption to workflow and relationships. They often made interim clinical plans and deferred help provision, rather than seeking in-consultation assistance. Although these portrayals may be influenced by social desirability pressures [33, 34], the authors gained the impression that the outcomes of help-seeking were usually positive for most trainees. Some of the negative outcomes reported appeared to be particularly ‘dramatic’ and atypical episodes [24, 25]. Our findings align with the subjective expected utility approach [8] and extend this approach by highlighting how trainee activities decrease any costs and/or maximise the benefits of help-seeking.

The trainee emphasis on patient safety by “*double-checking*” and not “*missing anything*” positions them as engaging in appropriate self-monitoring [22] for areas of unconscious incompetence [34–36] and the premature resolution of uncertainty [22, 23]. Trainees therefore portray themselves as safe, self-regulated practitioners rather than as anxious, risk-averse or over-dependent on their supervisors. Their framing of safe practice as *“being able to sleep at night”* alludes to the influence of anticipated regret [32, 33] and also suggests that trainees rapidly develop a strong sense of vocational identity [36, 37] and legitimacy as full general practitioners, personally responsible for the patients under their care.

There are some limitations of our study. Our focus group numbers are smaller than usually recommended, although ‘mini-focus groups’ and ‘paired interviews’ are established qualitative research methods [37–39]. Strengths of smaller groups include ease of recruitment, organisation and facilitation, and less fragmentation of discussion than larger groups [38, 39]. Limitations may include a restricted pool of ideas [38, 39]. However the groups were characterised by participant diversity, the absence of a power differential, and dynamic social interaction, all of which are important for the spontaneous articulation of different viewpoints [24, 37]. Our participants were largely metropolitan Australian trainees, and rural and/or international variations in arrangements for general practice trainee supervision [1, 39] may limit the transferability of our findings. Trainees may have consented to participate in our study because of particularly strong views or other reasons, potentially affecting the data collected and limiting transferability. We did not commence formal coding and analysis until data collection was complete (although the corresponding author facilitated every group, informally comparing and contrasting the data iteratively with each group, and we believe that we closely approached data saturation [40, 41]).

Strengths include the inclusion of diverse trainees across different stages of training (although the timing of data collection in association with end of year education release days resulted in Term 2 trainees being the largest group recruited), the use of focus groups, and our attention to complexity and inconsistency [28, 29] in the data, informed by existing literature and theory.

The ability to identify authoritative, readily accessible advice is an important skill for safe clinical practice [32, 41]. Trainees appeared to assess supervisor credibility in two key areas. The first was their approach to managing clinical risk, and trainees reported seeking help from supervisors whose approaches aligned most comfortably with their own. How trainees position themselves on this spectrum from cavalier to over-cautious may be an important aspect of their developing identity as general practitioners [42, 43]. The second area was their clinical expertise, and many trainees appeared to have the impression that this was patchy, and best approached by ‘cherry-picking’ the supervisor who seemed most knowledgeable in a particular clinical context, or on occasions seeking several ‘second’ opinions. From a community of practice perspective, some tension between old-timers and newcomers is expected, and trainee help-seeking is likely to play a role in both the transformation and reproduction of the community’s practice [11].

Help-seeking potentially affords trainees the valuable opportunity of observing, and learning to recognise, expert practice [43, 44], especially if supervisors interact directly with patients. However given the time constraints, and the limited information available, supervisors may tend to model simpler coping routines (such as briefly vetting trainee plans for safety) during both direct and indirect supervision, rather than more expert proficiency routines which engage with complexity [45, 46]. Trainees may therefore risk being excluded from this important affordance of workplace-based learning [12].

Trainees were somewhat ambivalent about the advice of supervisors to “*trust their judgement”*, although they appreciated validation from their supervisors and preferred to “*trust (their) gut*” rather than receive doubtful advice. Trainees gave the impression of accepting ongoing uncertainty and risk as part of becoming a general practitioner. Uncertainty may be particularly extensive, endemic and ineradicable in general practice [46, 47], although it is a complex concept [48], and further research is warranted to investigate trainee understanding, tolerance and management of uncertainty in their clinical practice.

The risks of ‘loss of face’ in front of patients when seeking help seem to be reduced by trainee use of “*second opinion*” terminology (which is also recommended to Australian general practitioner supervisors [7]). Several trainees portrayed themselves as comfortable seeking help in front of patients. However trainees remain vulnerable to exposure as lacking competence by supervisors with a poor understanding of the etiquette of help provision, who undermine trainee legitimacy [49, 50].

The frequency of in-consultation help-seeking seemed to vary considerably between trainees, after the initial transition into general practice when help-seeking appeared to be frequent. It would be interesting to know whether trainees with less in-consultation help-seeking use other self-regulated learning or help-seeking strategies more frequently. Further investigation of whether and how help-seeking influences trainee approaches to uncertainty, trainee appreciation of expert practice and disciplinary standards, and patient safety is also warranted.

## Conclusions

Implications for general practice supervision and supervisor training include the need for supervisors to be aware of the reluctance of trainees to disrupt practice workflow by seeking help, and the importance of developing supportive trainee-supervisor relationships, responding promptly to trainee requests, and observing the etiquette of help provision with patients. Trainees appear to learn rapidly through their help-seeking encounters to identify credible clinical advice and/or make their own clinical ‘calls’. However supervisors should continue to provide their trainees with opportunities to reflect together about the safe management of clinical uncertainty and other ‘grey areas’ of practice, and to observe expert general practitioners at work. Supervisors should encourage trainees to seek both in-consultation and deferred assistance throughout their training.

## Data Availability

The focus group transcript data will not be shared, as participant consent was not sought to make transcripts available outside the investigator team, and some data may identify participants or other parties.

## Abbreviations

FG: Focus group
GP: General practitioner
P: Participant
